# Dermoscopy of crusted lesion: diagnostic challenge and choice of technique for the analysis^☆^^☆☆^

**DOI:** 10.1016/j.abd.2020.06.016

**Published:** 2021-03-15

**Authors:** Agnes Carvalho Andrade, Marina Zoéga Hayashida, Milvia Maria Simões e Silva Enokihara, Sérgio Henrique Hirata

**Affiliations:** Department of Dermatology, Universidade Federal São Paulo, São Paulo, SP, Brazil

Dear Editor,

A 52-year-old male patient, phototype IV, with significant photodamage complained of a painful chest lesion, with a progressive increase in the 6 previous months. During the physical examination, he had an erythematous plaque measuring 4 × 8 cm, with yellowish crusts and violaceous borders on the left anterosuperior thorax region (Fig. 1). Dermoscopy was inconclusive due to the presence of nonspecific yellowish crusts on the lesion (Fig. 2).

**Figure 1:**
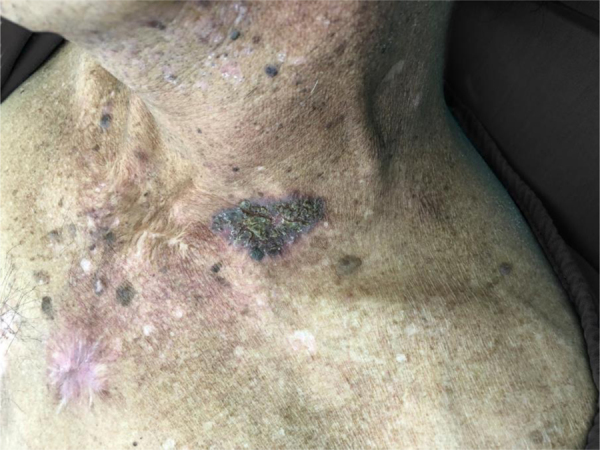
Desquamative plaque on the anterosuperior thorax.

**Figure 2:**
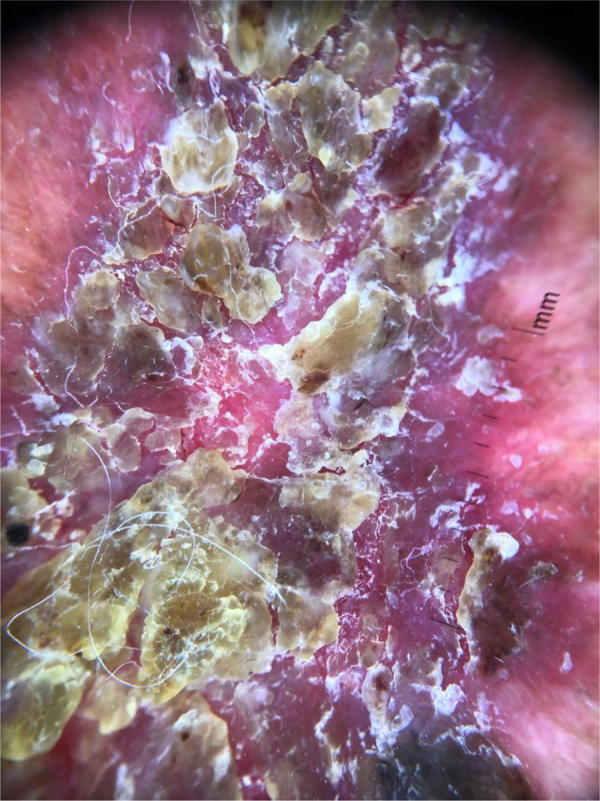
Dermoscopy before removal of crusts.

After applying wet compresses with saline solution, the crusts were carefully removed. To avoid contact with the lesion surface, polarized light dermoscopy was performed, which demonstrated the presence of glomerular vessels, suggestive of Bowen's disease (BD) (Fig. 3).^1^ In view of this picture, an excisional biopsy was performed. Histopathological examination confirmed the diagnosis of BD.

**Figure 3:**
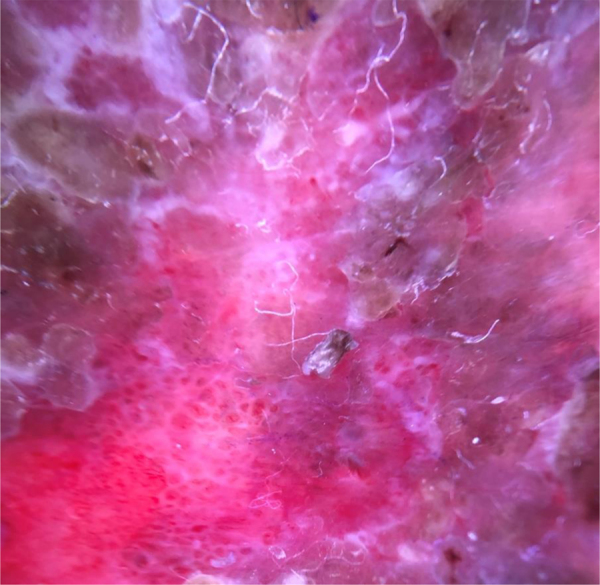
Dermoscopy after removal of crusts, displaying glomerular vessels.

Dermoscopy is important in the diagnosis of pigmented and non-pigmented lesions.^1^ In non-pigmented lesions, the vascular pattern can lead to the diagnosis, but in the reported case, it was not possible to achieve the diagnosis due to the presence of crusts. The presence of yellowish crusts at dermoscopy has been previously described in 78.8% of 146 evaluated BD lesions, but it is not specific, and the presence of crusts alone is not a sufficient criterion for this diagnosis.^2^, ^3^ In crusted lesions, the removal of the crusts may allow the observation of other dermoscopic structures that can help achieve the diagnosis. The removal of crusts should be performed with caution to avoid damage to the epithelium because this maneuver can allow the observation of other dermoscopic structures that make the diagnosis possible.

## Financial support

None declared.

## Authors’ contributions

Agnes Carvalho Andrade: Approval of the final version of the manuscript; study design and planning; drafting and editing of the manuscript; collection, analysis, and interpretation of data; effective participation in the research orientation; critical review of the literature; critical review of the manuscript.

Marina Zoéga Hayashida: Approval of the final version of the manuscript; study design and planning; drafting and editing of the manuscript; effective participation in research orientation.

Milvia Maria Simões e Silva Enokihara: Approval of the final version of the manuscript; drafting and editing of the manuscript; effective participation in the research orientation; critical review of the literature; critical review of the manuscript.

Sérgio Henrique Hirata: Approval of the final version of the manuscript; study design and planning; drafting and editing of the manuscript; collection, analysis, and interpretation of data; effective participation in the research orientation; intellectual participation in the propaedeutic and/or therapeutic conduct of the studied cases; critical review of the literature.

## Conflicts of interest

None declared.
